# Predictive Modeling of Acute Hypertensive Disorders in a Real-World Cohort: Integrating Clinical Predictors and Data-Driven Methods

**DOI:** 10.3390/diagnostics15162062

**Published:** 2025-08-18

**Authors:** Ilaria Fucile, Filomena Liccardi, Maria Virginia Manzi, Maria Lembo, Christian Basile, Orlando Santucci, Stefania Auciello, Mauro Maniscalco, Giorgio Alfredo Spedicato, Carmine Morisco, Raffaele Izzo, Nicola De Luca, Pasquale Ambrosino, Costantino Mancusi, Giovanni Esposito, Fiorella Paladino

**Affiliations:** 1 Hypertension Research Center, Department of Advanced Biomedical Science, Federico II University, 80131 Naples, Italy; fucile.ilaria@gmail.com (I.F.); mariavirginia.manzi@unina.it (M.V.M.); maria.lembo@unina.it (M.L.); santucciorlando@gmail.com (O.S.); carmine.morisco@unina.it (C.M.); raffaele.izzo@unina.it (R.I.); nideluca@unina.it (N.D.L.); espogiova@unina.it (G.E.); 2 Emergency Department, Antonio Cardarelli Hospital, 80131 Naples, Italy; liccardif@gmail.com (F.L.); astefaniamichela@gmail.com (S.A.); fiorepaladino@gmail.com (F.P.); 3 Department of Clinical Science and Education, Karolinska Institutet, 171 77 Stockholm, Sweden; christian.basile.94@gmail.com; 4 ANMCO Research Center, Heart Care Foundation, 50121 Florence, Italy; 5 Istituti Clinici Scientifici Maugeri IRCCS, Pulmonary Rehabilitation Unit of Telese Terme Institute, 82037 Telese Terme, Italy; mauro.maniscalco@icsmaugeri.it; 6 Department of Clinical Medicine and Surgery, Federico II University, 80131 Naples, Italy; 7 Department of Statistics and Quantitative Methods, Milano-Bicocca University, 20126 Milan, Italy; spedicato_giorgio@yahoo.it; 8 Istituti Clinici Scientifici Maugeri IRCCS, Scientific Directorate of Telese Terme Institute, 82037 Telese Terme, Italy

**Keywords:** hypertension, biomarkers, machine learning, disability, rehabilitation, cardiovascular risk, chronic disease, outcome

## Abstract

**Background**: Acute hypertensive disorders, including hypertensive emergencies (HEs) and urgencies (HUs), are a frequent cause of emergency department (ED) visits. Early differentiation between HEs and HUs is essential, as their clinical management and prognostic implications differ substantially. **Methods**: We retrospectively analyzed patients admitted to an Italian second-level ED between January and June 2022 with systolic blood pressure (SBP) ≥ 180 mmHg and/or diastolic blood pressure(DBP) ≥ 110 mmHg. Patients were categorized based on the presence of acute hypertension-mediated organ damage (A-HMOD). To identify the main predictors of HEs, we applied both conventional logistic regression and machine learning approaches (Elastic Net and Random Forest). **Results**: Among 23,678 ED admissions, 261 patients (1.1%) had acute hypertensive disorders, of whom 115 (44%) were diagnosed with HEs and 146 (56%) with HUs. Compared with HU patients, HE patients were older and showed higher SBPand DBP at presentation, along with a greater prevalence of comorbidities such as diabetes, coronary artery disease, and chronic kidney disease (all *p* < 0.05). In multivariable logistic regression, troponin I levels independently predicted the occurrence of HEs (OR: 2.82; 95%CI: 1.65–4.82; *p* < 0.001), even after adjusting for confounders. Machine learning analyses confirmed troponin I as the most influential predictor, followed by age and SBP, with the Random Forest model achieving a high predictive performance (AUC_ROC_: 0.93; 95%CI: 0.90–0.96). Elastic Net regression further highlighted troponin I as the most influential variable with the highest standardized coefficient (β = 4.13). As determined by the Youden index, the optimal diagnostic threshold for troponin I was 0.12 ng/mL (AUC_ROC_: 0.66; 95%CI: 0.60–0.72). **Conclusions**: In patients presenting to the ED, withacute hypertensive disorders, elevated troponin I levels, older age, and higher SBP at admission may serve as early indicators of emergencies.

## 1. Introduction

An acute elevation in blood pressure (BP) is a common cause of admission to the emergency department (ED). Despite widespread awareness of its associated high morbidity and mortality, BP control rates remain suboptimal globally and are unsatisfactory across Europe [1,2]. Consequently, hypertension remains the major preventable cause of cardiovascular disease (CVD) and all-cause mortality globally [3,4]. The severity of an acute hypertensive disorder is determined not only by the absolute level of BP, but also by the presence of signs of acute organ damage, which distinguish hypertensive emergencies (HEs) from hypertensive urgencies (HUs) [5].

HEs are clinical conditions in which a severely elevated BP, usually a systolic blood pressure (SBP) greater than 180 mmHg and/or a diastolic blood pressure (DBP) greater than 110 mmHg, is associated with acute hypertension-mediated organ damage (A-HMOD) [6]. This condition may present with encephalopathy, cerebral ischemia or hemorrhage, acute coronary syndrome, myocardial dysfunction with pulmonary edema, aortic dissection, or acute kidney injury [7]. Conversely, cases of severely elevated BP without A-HMOD are classified as HUs.

Acutely increased BP may occur de novo in patients with no known history of hypertension and in those with untreated and/or uncontrolled hypertension, with the latter being more common. Secondary causes of hypertension, including renovascular hypertension, renal parenchymal disease, endocrine disorders, drug abuse, concomitant medications, and pheochromocytoma, may lead to hypertensive crises more frequently than spontaneous events in patients with essential hypertension [8].

A major challenge for emergency clinicians is the prompt recognition and management of HEs, due to their different clinical presentations and significant risk of adverse cardiovascular and cerebrovascular outcomes. HEs require a rapid and carefully controlled reduction in BP, typically within minutes or a few hours, to reverse or limit A-HMOD. Treatment choice, BP targets, and the timing of BP reduction depend on the specific type of hypertensive organ damage. However, all patients should be admitted to an intensive care unit for close BP monitoring and administration of appropriate intravenous drugs [9].

Conversely, patients with HUs typically do not require hospitalization, but rather only a brief period of observation. Nevertheless, BP reduction is always necessary and can be achieved through the oral administration of antihypertensive drugs, aimed at gradually lowering BP over 24 to 48 h. Oral treatment may involve the reinstitution or intensification of previous treatment or the initiation of a new regimen [3]. Overall, the management of acute hypertensive crises across various care settings (community, hospital, rehabilitation) and the prompt differentiation between HUs and HEs are crucial, given the distinct therapeutic approaches and the different short- and long-term outcomes associated with each condition [10,11].

The aim of this study was to assess the incidence rate of acute hypertensive disorders among cases presenting to a second-level ED, with a focus on patient characteristics, treatment strategies, and clinical outcomes. Additionally, advanced analytical models were employed to identify the main predictors of A-HMOD in this real-world ED setting, with the goal of informing future predictive models capable of distinguishing HEs from HUs.

## 2. Methods

We conducted a retrospective chart review of patients over 18 years of age who were admitted to the ED of “Antonio Cardarelli” Hospital in Naples, Italy, with an SBP ≥ 180 mmHg and/or a DBP ≥ 110 mmHg between January and June 2022.

Patients were classified as having either HEs or HUs based on the diagnosis of A-HMOD, as documented in the final discharge diagnoses recorded in the electronic medical records. A-HMOD was defined as the presence of one or more of the following: hypertensive encephalopathy, ischemic stroke, intracerebral hemorrhage, subarachnoid hemorrhage, acute heart failure, acute coronary syndrome, or aortic dissection. Following our institutional ED protocol, end-organ damage was assessed through laboratory tests, electrocardiography (ECG), and imaging studies including chest X-ray, computed tomography (CT), and ultrasound examinations. In brief, stroke (ischemic or hemorrhagic) and subarachnoid hemorrhage were confirmed by brain CT according to American Heart Association/American Stroke Association guidelines [12]. Acute heart failure was diagnosed based on clinical signs and symptoms (e.g., dyspnea, pulmonary rales), radiological evidence of pulmonary congestion on chest imaging, and elevated natriuretic peptides when available, in accordance with the 2021 European Society of Cardiology guidelines [13]. Acute coronary syndrome was defined by the presence of typical chest pain, ischemic ECG changes, and elevated troponin I above the institutional upper reference limit [14]. Aortic dissection was confirmed by contrast-enhanced CT angiography [15], while hypertensive encephalopathy was diagnosed in the presence of acute neurological symptoms, compatible neuroimaging findings, and the exclusion of alternative etiologies [9]. For the purposes of this study, neurological symptoms included acute vertigo, focal neurological deficits, and altered consciousness characterized by a reduced ability to focus, sustain, or shift attention. We excluded women with eclampsia, preeclampsia, or hemolysis, elevated liver enzymes, and low platelet count (HELLP) syndrome, as they were directly referred to the Obstetrics Department, as well as patients who tested positive for severe acute respiratory syndrome coronavirus 2 (SARS-CoV-2) on rapid testing in triage, as they were managed through a dedicated pathway. Additionally, patients with an elevated BP due to trauma or known pain were excluded.

This study was conducted in compliance with the Strengthening the Reporting of Observational Studies in Epidemiology (STROBE) guidelines [16], and in accordance with the Declaration of Helsinki of the World Medical Association (WMA). The protocol (No. 04/25, 05 March 2025) was reviewed and approved by the Institutional Review Board of Comitato Etico Territoriale Campania 3.

### 2.1. Study Variables

Data collected and analyzed included age, sex, pertinent medical history (such as hypertension, diabetes mellitus, chronic kidney disease [CKD], and coronary artery disease [CAD]), presenting symptoms at admission, antihypertensive therapy administered in the ED, and findings from biochemical and radiological investigations. Additionally, data on patient discharge or hospitalization and in-hospital mortality were recorded.

### 2.2. Study Procedures

BP was measured with the patient in either a sitting or supine position using validated automatic sphygmomanometers, in accordance with current guidelines [3]. For patients with HU, BP was measured both in triage and in the ED room where the examination was conducted. In patients with HE, BP was measured, in most cases, directly in the red code room as they were transported by ambulance. Therefore, to ensure consistent data collection, only BP measurements obtained by members of the same medical team using the same equipment in the ED were considered.

The estimated glomerular filtration rate (eGFR) was calculated using the Chronic Kidney Disease Epidemiology Collaboration (CKD-EPI) formula, based on serum creatinine levels measured in the ED. An eGFR of less than 60 mL/min/1.73 m^2^ was considered indicative of chronic kidney disease.

Troponin I was measured using a conventional immunoassay with a detection limit of 0.10 ng/mL. The laboratory-defined upper reference limit for normal values was 0.30 ng/mL. Given the detection threshold, our assay did not meet the criteria for a high-sensitivity troponin test. Consequently, the lowest reported troponin I value in this study was 0.10 ng/mL.

### 2.3. Statistical Analysis

Statistical analyses were performed with the IBM SPSS Statistics 22.0 system (Chicago, IL, USA) and R-4.5.1 software (R Core Team 2025). Continuous variables were expressed as mean ± standard deviation (SD) if normally distributed or median (interquartile range, IQR) in case of non-normal distribution. The Shapiro–Wilk test was used to assess whether the sample was generated from a normal distribution. Student’s *t*-test for unpaired samples was used for homogeneous variances, otherwise Welch’s *t*-test was employed. Whenever applicable, a Mann–Whitney *U*-test for unpaired samples was also used in case of non-normal distribution. Categorical variables were reported as absolute and percentage frequencies and the Pearson’s χ^2^ test was used for comparison. To facilitate predictions, binomial logistic regression analyses were conducted, calculating the odds ratios (ORs) to quantify the strength of the associations.

In addition to descriptive statistics and a multivariate logistic regression model, machine learning approaches were also applied to improve classification performance and assess variable importance. In particular, the Elastic Net (EN) method, combining Lasso and Ridge regularization, was used to address multicollinearity and perform variable selection, with model relevance determined by the magnitude of standardized coefficients (β). Moreover, the Random Forest (RF) algorithm, a non-parametric ensemble method based on bootstrapped decision trees, evaluated predictor importance through the mean decrease in Gini impurity. In addition, information gain (IG), an entropy-based metric, was computed for all features to quantify how much each variable contributed to reducing uncertainty in classification. A stratified 5-fold cross-validation was used on both the EN and RF models to avoid overfitting and assess their predictive performance. Finally, for key predictors identified in multivariate models, receiver operating characteristic (ROC) analysis was used to determine the optimal classification threshold, defined by the point maximizing Youden’s index.

A *p*-value < 0.05 was considered statistically significant and no imputation methods were applied for any of the analyses. Descriptive statistics and logistic regressions were based on available complete cases, while the RF model handled missing data through internal surrogate splits.

## 3. Results

**Figure 1:**
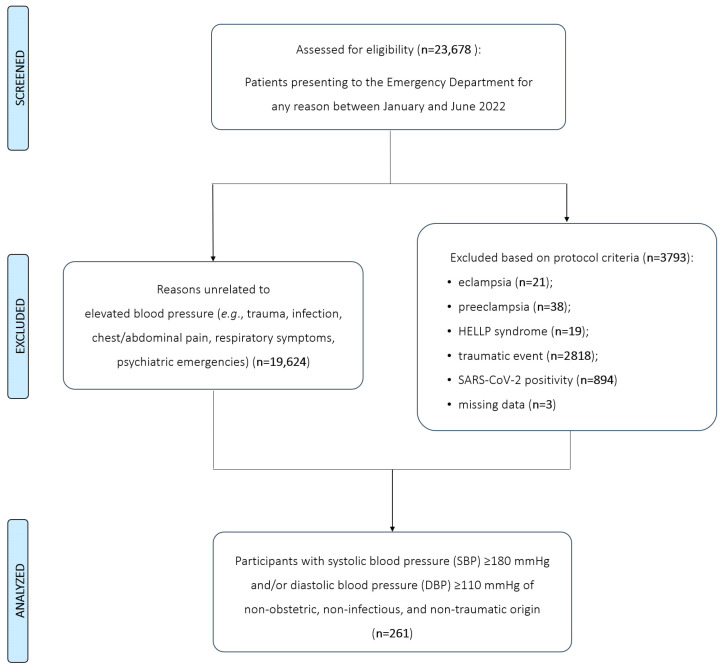
Strengthening the Reporting of Observational Studies in Epidemiology (STROBE) flow diagram of study participants presenting to the emergency department (ED) with acute hypertensive disorders. Abbreviations: n—number; HELLP—hemolysis, elevated liver enzymes, and low platelet count; SARS-CoV-2—severe acute respiratory syndrome coronavirus 2.

Starting from a cohort of 23,678 individuals who accessed our ED during the study period, 3790 were excluded due to a diagnosis of eclampsia, preeclampsia, or HELLP syndrome; a concomitant traumatic event; or a positive SARS-CoV-2 rapid assay at triage. An additional 19,624 patients presented to the ED for reasons unrelated to elevated BP, including trauma, infections, abdominal or chest pain, respiratory symptoms, psychiatric emergencies, and other non-hypertensive conditions. Thus, during the six-month study period, acute hypertensive disorders of non-obstetric, non-infectious, and non-traumatic origin were diagnosed in 264 individuals, corresponding to an incidence rate of 1.1%. However, three patients were excluded due to a high proportion of missing data relevant to the study objectives, specifically the lack of sufficient clinical information to reliably differentiate between HE and HU at presentation. As a result, 261 patients had sufficient data for analysis, including 115 (44%) with HE and 146 (56%) with HU (Figure 1).

### 3.1. Demographic and Clinical Characteristics

The baseline characteristics of the patients are shown in Table 1. The mean age was 68 years, with 58% being male and 76.4% having a known history of hypertension. Other relevant comorbidities included diabetes mellitus in 25.8% of patients, CKD in 8.8%, and CAD in 21.2%. At the time of ED presentation, the median SBP was 187 mmHg (IQR: 180.0–200.0), and the median DBP was 100 mmHg (IQR: 90.0–110.0). Nearly all patients underwent at least basic laboratory testing and ECG, over half received a chest X-ray or CT, 36% underwent brain CT imaging, while cardiology consultation was required in 31.8% of patients. Following pharmacological treatment with various classes of antihypertensive agents, approximately half of the patients were admitted to inpatient wards, while 36.4% were discharged directly from the ED. There were 10 deaths (3.8%), all in the HE group, with the remaining patients either discharged against medical advice or managed in a short stay observation unit.

**Figure 2:**
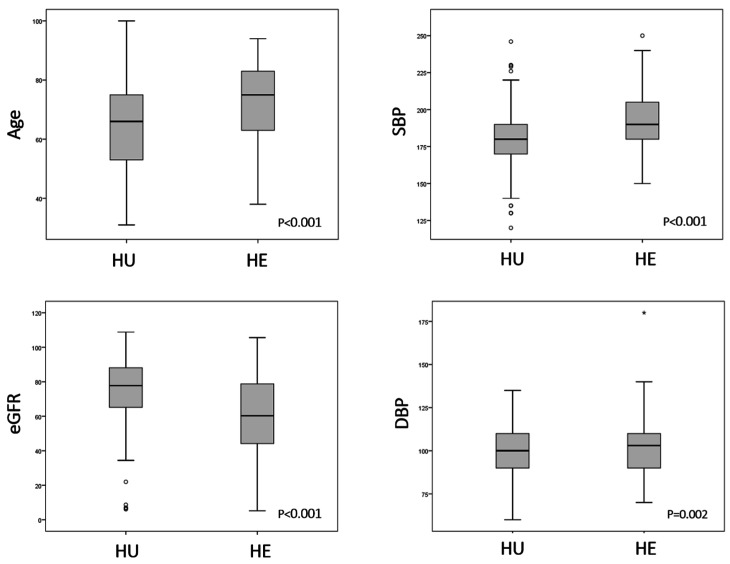
Age, systolic blood pressure (SBP), diastolic blood pressure (DBP), and estimated glomerular filtration rate (eGFR) in patients with hypertensive urgency (HU) and emergency (HE).

As illustrated in Figure 2, some relevant differences emerged when comparing patients with HU and those with HE. Specifically, the HE patient group was significantly older, with a median age of 75.0 years (IQR: 63.0–83.0) compared with 66.0 years (IQR: 53.0–75.3) in the HU group (*p* < 0.001). Upon ED presentation, the HE patients also exhibited a significantly higher median SBP (190.0 vs. 180.0 mmHg, *p* < 0.001) and DBP (103.0 vs. 100.0 mmHg, *p* = 0.002) compared with the HU patients. While the prevalence of known treated hypertension was similar between the two groups (approximately 76%), the HE patients demonstrated a significantly higher prevalence of diabetes mellitus (35.7% vs. 17.9%, *p* = 0.002), CAD (27.8% vs. 16.0%, *p* = 0.030), and CKD (14.8% vs. 4.1%, *p* = 0.005). Consistent with these findings, the HE patients also had significantly higher median serum creatinine (1.06 vs. 0.85 mg/dL, *p* < 0.001) and lower eGFR values (60.3 vs. 77.8 mL/min/1.73 m^2^, *p* < 0.001).

**Figure 3:**
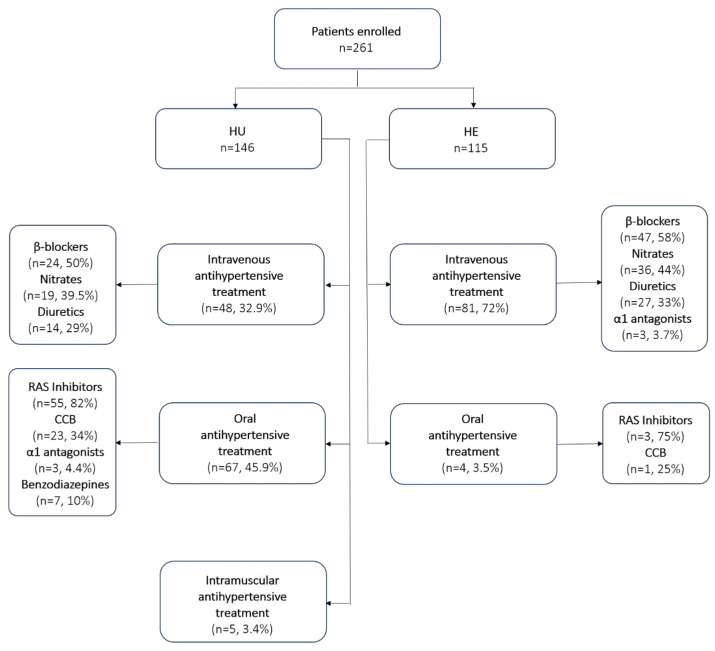
Age, systolic blood pressure (SBP), diastolic blood pressure (DBP), and estimated glomerular filtration rate (eGFR) in the emergency department (ED) among patients with acute hypertensive disorders. Abbreviations—HE—hypertensive emergency; HU—hypertensive urgency.

Half of the patients with HU were asymptomatic at the time of evaluation, with nonspecific symptoms observed in the remaining cases, the four main clinical manifestations of A-HMOD among HE patients were acute coronary syndrome, ischemic stroke, hemorrhagic stroke, and pulmonary edema, each occurring in approximately one quarter of the HE population and leading to immediate hospitalizations in most cases (93%). As expected, antihypertensive treatment in the HE group was predominantly administered intravenously, significantly more often than in HU patients (70.4% vs. 32.9%, *p* < 0.001), whereas oral administration was more frequently employed in HUs (45.9% vs. 3.5%, *p* < 0.001). Differences were also observed in the classes of antihypertensive drugs prescribed, with renin–angiotensin system (RAS) inhibitors being more commonly used in HU patients (33.7% vs. 2.6%, *p* < 0.001), while nitrates were more frequently administered to participants with HE (31.3% vs. 13.0%, *p* = 0.001) (Figure 3).

### 3.2. Multivariable and Machine Learning Analysis of A-HMOD Predictors

A standard binomial logistic regression was performed to ascertain the effects of age, sex, SBP, DBP, heart rate, diabetes, known treated hypertension, CKD, CAD, creatinine, eGFR, and troponin levels on the likelihood of participants presenting with HE. The logistic regression model was statistically significant, χ^2^(12) = 66.114, *p* < 0.001. The model explained 38.7% (Nagelkerke R^2^) of the variance in the dependent variable and correctly classified 72.7% of cases. Of the predictor variables, only two were statistically significant. Specifically, an increasing age (OR: 1.043; 95%CI: 1.006, 1.081; *p* = 0.023) and SBP (OR: 1.021; 95%CI: 1.002, 1.041; *p* = 0.032) were associated with an increased likelihood of HE. However, considering the troponin I detectability threshold of 0.10 ng/mL and the clinical upper reference limit of 0.30 ng/mL established by our laboratory, we stratified participants into three groups according to their troponin levels: ≤0.10 ng/mL, >0.10 to <0.30 ng/mL, and ≥0.30 ng/mL.

This categorical troponin variable was thus included in a revised regression model and, while the results for age (OR: 1.043; 95%CI: 1.006–1.081; *p* = 0.022) and SBP (OR: 1.022; 95%CI: 1.003–1.041; *p* = 0.021) remained substantially unchanged (Nagelkerke R^2^ = 35.5%), troponin category emerged as the strongest independent predictor of HE, with a stepwise increase in odds across categories (OR: 2.819; 95%CI: 1.649–4.820; *p* < 0.001) (Table 2).

**Figure 4:**
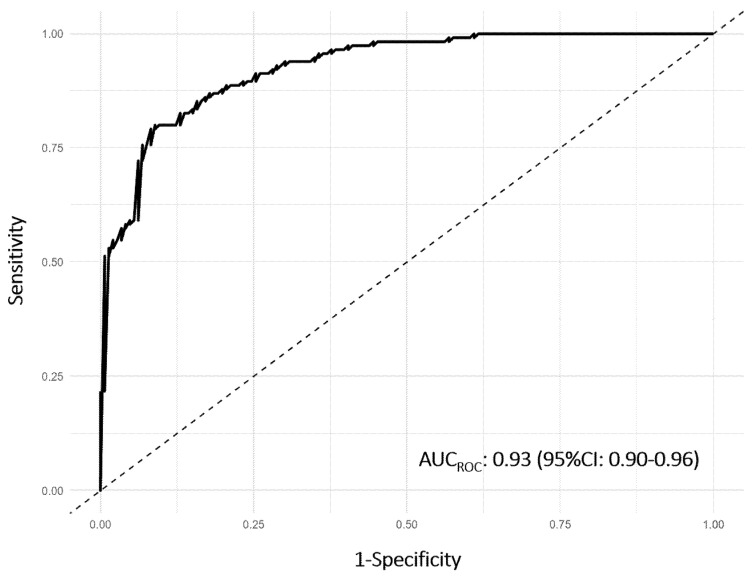
Area under the receiver operating characteristic (AUC_ROC_) curve with 95% confidence interval (95%CI) of Random Forest (RF) algorithm.

To complement the ordinary regression analysis, a machine learning approach using a RF classifier was also applied, confirming troponin I (IG: 10.22%), age (IG: 7.80%), and SBP (IG: 8.04%) as the most relevant predictors of A-HMOD (Table 2). Overall, the model exhibited excellent predictive performance, with an area under the ROC (AUC_ROC_) curve of 0.93 (95%CI: 0.90–0.96) (Figure 4). An additional analysis using an EN logistic regression further supported the central role of troponin I as the strongest predictor of HE. Among all variables included in the model, troponin I had by far the highest standardized coefficient (β = 4.13), indicating a strong positive association with the outcome. Interpreted on the odds scale, this corresponds to an OR of approximately 62.2 (OR: e^.4.13^), suggesting that, holding other variables constant, each one SD increase in troponin level was associated with more than a 60-fold increase in the odds of HE. Furthermore, the optimal diagnostic threshold for troponin I, as determined by the Youden index, was identified at 0.12 ng/mL, corresponding to an AUC_ROC_ of 0.66 (95%CI: 0.60–0.72), indicating moderate discriminatory ability at this specific cut-off (Supplemental Figure S1).

## 4. Discussion

With an incidence rate of 1.1% among all visits to our second-level ED over a six-month period, acute hypertensive disorders represent a relevant healthcare burden, contributing to increased resource utilization, a higher risk of severe cardiovascular events, and significant pressure on emergency services. Our findings confirm and expand upon the existing literature, particularly meta-analytic data [17] suggesting that patients with HU tend to be younger and more frequently present either asymptomatically or with nonspecific symptoms such as headache. Our study also reinforces the observation that patients with HE exhibit a higher burden of metabolic dysfunction and comorbidities, more often presenting to the ED with symptoms indicative of acute organ damage, such as dyspnea, focal neurological deficits, and altered consciousness. However, it is important to note that even HE cases can initially present with nonspecific symptoms, especially in the early phase [18,19]. Therefore, the prompt recognition and differentiation between HEs and HUs remains essential to guide appropriate treatment and to define a reliable prognostic framework. By employing advanced analytical methods beyond traditional statistics and relying on a moderately sized dataset, our study identified increasing troponin I levels, age, and SBP at ED presentation as the most relevant warning indicators of HE. These variables may therefore be suitable candidates for inclusion in future predictive models for A-HMOD.

Although the role of age as a predictor of organ damage risk in hypertensive patients is well established and, to some extent, expected [20], the role of SBP is more surprising, given that meta-analytic data have suggested that BP values alone may not reliably distinguish between patients with HE and those with HU [17]. However, our findings do not contradict this notion, but rather indicate that a statistically significant difference may exist, with a median SBP of 190 mmHg in HE versus 180 mmHg in HU, suggesting that particularly high SBP values should perhaps receive greater clinical attention. Similarly, the role of troponin I as the strongest predictor of A-HMOD is not entirely surprising, given that nearly one third of our HE patients were diagnosed with acute coronary syndrome. However, not all participants in our study with values above the normal cut-off defined by our laboratory (0.30 ng/mL) had a myocardial infarction, whereas the diagnostic threshold identified for A-HMOD in our analyses (0.12 ng/mL) remained well below this limit. In fact, although troponin I is more specific to cardiac muscle than troponin T and is less affected by renal function [21,22], its elevation does not necessarily reflect overt myocardial injury, potentially indicating even subclinical cardiac involvement or increased myocardial strain due to increased afterload [23]. Consistent with our findings, a post hoc analysis of the SPRINT trial showed a greater decrease in troponin I levels with intensive BP control, along with the robust prognostic value of longitudinal changes in troponin I irrespective of antihypertensive treatment [21]. It is important to note that in our study we did not use a high-sensitivity troponin I assay but rather a conventional assay with a lower detection limit of 0.10 ng/mL. Although, as discussed below, this represents an absolute limitation in terms of sensitivity; it also strengthens the clinical validity of our findings, as troponin I emerged as a strong predictor of A-HMOD despite the inability to detect subtle elevations. Nevertheless, it should be acknowledged that high-sensitivity troponin assays are now widely used in clinical practice, as they allow for the detection of even subtle increases above baseline, indicating not only subclinical myocardial injury but also other acute pathologies such as pulmonary embolism, ischemic or hemorrhagic stroke, sepsis, acute kidney injury, and major bleedings [24]. It is interesting to note how many of these conditions fall within the spectrum of A-HMOD, further supporting the hypothesis that high-sensitivity troponin could further enhance the predictive value of this biomarker in the setting of acute hypertensive disorders. Future studies should now explore whether the application of these high-sensitivity assays may effectively improve risk stratification and predictive accuracy in this clinical context.

It is evident that our findings cannot be generalized, as they reflect the experience of a single second-level ED in the Campania region of Italy and involve a population with a higher clinical severity. In this regard, while the existing literature based on settings and sample sizes similar to ours reports that approximately 27.5% of hypertensive crises are HEs [25], in our cohort this proportion rises to 44%, with this difference being explained by the fact that our hospital serves as a referral center for both percutaneous coronary intervention and stroke care. On the other hand, with over 76% of patients in both the HU and HE groups having a known history of hypertension, our cohort remains representative of a clinical scenario in which poor adherence to antihypertensive therapy appears to be the primary driver of acute BP elevations [25,26,27], even among patients who self-report full adherence to their prescribed treatment [28]. The fact that as many as 23.8% of our patients were asymptomatic further underscores the real-life value of our study, especially in light of current guidelines [29,30,31], which discourage referring patients with HUs to the ED and instead recommend initiating or adjusting antihypertensive therapy in the same setting where the patient is being managed, whether at home, in primary care, or in a rehabilitation facility [32,33,34]. Therefore, our findings reinforce the importance of early differentiation between HEs and HUs, given that presenting symptoms are not always sufficient to guide appropriate management. Chest pain, for instance, was among the most frequently reported in our sample, affecting approximately one quarter of patients in both the HU and HE groups. It is noteworthy how chest pain is one of the most common yet diagnostically complex reasons for ED visits [35], and in many cases, its origin is non-cardiac and non-urgent, often related to musculoskeletal or gastrointestinal causes [36]. The results of our regression models, supported by machine learning analysis, strongly highlight the role of troponin I as a predictor of A-HMOD. This finding is consistent with its established use in the diagnostic evaluation of chest pain and acute coronary syndrome [36], as well as ischemic stroke [12] and acute heart failure [37]. Prospective studies are now needed to determine whether the predictors identified in our analysis can support the development of prognostic models for disabling hypertensive complications, as already conducted for other clinical conditions [38,39,40].

### Limitations

This study has some important limitations. One of the most relevant limitations is its retrospective design, which inherently limits the generalizability of our findings. In particular, selection bias may have influenced our results, as suggested by the unusually high proportion of HEs observed among patients presenting with a hypertensive crisis (nearly half in our cohort). Conversely, it is worth noting that our study reflects a real-world clinical scenario, with the robustness of its findings further supported by a relatively large sample size and minimal missing data, particularly at a time when even randomized controlled trials are increasingly subject to critical evaluation [41].

Another important consideration concerns our attempt to adopt a more advanced analytical approach. Although our dataset cannot be classified as small, its size is not sufficiently large for the application of more computationally intensive machine learning techniques. For this reason, we opted for RF, which is generally more robust when dealing with moderate-sized datasets. Moreover, the EN method was also selected for its efficacy in managing situations characterized by a relatively high number of predictor variables compared with the number of observations. Combining Ridge and Lasso regularizations, the EN method is advantageous as it penalizes and potentially excludes non-influential or highly collinear predictors, thereby enhancing model parsimony. Despite the limited size of our dataset for machine learning, which precludes definitive conclusions, it is noteworthy that the application of EN and RF, alongside conventional statistical models, provided complementary perspectives on variable relevance in predicting HE. The convergence of all three approaches in identifying troponin I as the most important predictor further strengthens the consistency and robustness of our findings regarding variable importance.

An additional limitation of our study lies in the assay used for troponin I measurement, which was a conventional assay rather than a high-sensitivity one, with a lower detection limit of 10 ng/mL. As a result, when analyzing troponin I as a continuous variable, the lower bound of the IQR coincided with the median value, indicating that more than half of the patients had troponin I levels at or below 10 ng/mL. While this likely inflated the observed troponin I values and constitutes an inherent limitation of our measurements, it also strengthens the reliability of our findings by highlighting the predictive value of troponin I even in the absence of high-sensitivity detection. Our RF models accounted for this limitation, as they naturally handle the relationship between the outcome and the predictors in a non-linear way. Furthermore, when troponin I was incorporated into our regression models as a categorical variable by defining three distinct levels rather than treating it as continuous, the troponin category emerged as the most powerful independent predictor of HE, with a stepwise increase in odds across categories. Therefore, we are confident that the analytical strategies employed allowed us to partially overcome this methodological limitation, further validating the role of troponin I as a critical predictor.

## 5. Conclusions

Our findings suggest that, with an incidence rate of 1.1% among all ED visits, acute hypertensive disorders may constitute a significant healthcare burden. Given the distinct therapeutic approaches required and current recommendations to manage uncomplicated HUs in outpatient settings, it is now critically important to develop predictive models capable of identifying HEs early, before overt signs of target organ damage become apparent. In this context, advanced analytical methods highlighted rising troponin I levels, older age, and higher SBP at presentation as the most relevant early warning indicators of HEs, supporting their potential inclusion in future predictive tools for A-HMOD.

## Figures and Tables

**Table 1:** Clinical and demographic characteristics of the study cohort with acute hypertensive disorders who presented to the emergency department during the study period.

Variable	Total Population	HE	HU	*p* Value
	261	115	146	
**Demographic**				
Age, Years	69.0 (57.5–79.0)	75.0 (63.0–83.0)	66.0 (53.0–75.3)	**<0.001**
Males, %	151 (57.9)	72 (62.6)	79 (54.1)	0.210
**Comorbidities**				
Hypertension, *n* (%) *(n = 259)*	198 (76.4)	88 (76.5)	110 (76.4)	1.000
Diabetes Mellitus, *n* (%) *(n = 260)*	67 (25.8)	41 (35.7)	26 (17.9)	**0.002**
Chronic Kidney Disease, *n* (%) *(n = 260)*	23 (8.8)	17 (14.8)	6 (4.1)	**0.005**
Coronary Artery Disease, *n* (%) *(n = 259)*	55 (21.2)	32 (27.8)	23 (16.0)	**0.030**
**Clinical parameters in the ED**				
SBP, mmHg	187.0 (180.0–200.0)	190.0 (180.0–210.0)	180.0 (170.0–201.0)	**<0.001**
DBP, mmHg	100.0 (90.0–110.0)	103.0 (90.0–110.0)	100.0 (90.0–110.0)	**0.002**
Heart Rate, bpm *(n = 217)*	81.0 (71.0–100.0)	85.5 (75.3–110.0)	80.0 (70.0–90.0)	**0.007**
**Laboratory parameters in the ED**				
Troponin I, ng/mL *(n = 233)*	0.10 (0.10–0.14)	0.10 (0.10–0.23)	0.10 (0.10–0.10)	**<0.001**
Creatinine, mg/dl *(n = 240)*	0.95 (0.77–1.20)	1.06 (0.86–1.42)	0.85 (0.75–1.10)	**<0.001**
eGFR, ml/min/1.73 m^2^ *(n = 240)*	72.0 (53.4–83.5)	60.3 (44.0–79.2)	77.8 (65.1–88.2)	**<0.001**
**Clinical Manifestations in the ED**				
Chest pain, *n* (%)	62 (23.8)	29 (25.2)	33 (22.6)	0.729
Dyspnea, *n* (%)	51 (19.5)	35 (30.4)	16 (11.0)	**<0.001**
Headache, *n* (%)	38 (14.6)	6 (5.2)	32 (21.9)	**<0.001**
Neurological Disorders, *n* (%)	65 (24.9)	49 (42.6)	16 (11.0)	**<0.001**
Epistaxis, *n* (%)	5 (1.9)	1 (0.9)	4 (2.7)	0.523
Asymptomatic, *n* (%)	62 (23.8)	1 (0.9)	61 (41.8)	**<0.001**
Pulmonary edema, *n* (%)	30 (11.5)	30 (26.1)	-	-
Acute Coronary Syndrome, *n* (%)	33 (12.6)	33 (28.7)	-	-
Ischemic Stroke, *n* (%)	27 (10.3)	27 (23.5)	-	-
Hemorrhagic Stroke, *n* (%)	23 (8.8)	23 (20.0)	-	-
Aortic Dissection, *n* (%)	2 (0.8)	2 (1.7)	-	-
**Diagnostics in the ED**				
Laboratory, *n* (%)	241 (92.3)	115 (100)	126 (86.3)	**<0.001**
ECG, *n* (%)	261 (100)	115 (100)	146 (100)	**-**
Abdominal Ultrasound, *n* (%)	34 (13.0)	9 (7.8)	25 (17.1)	**0.027**
Chest X-Ray or CT, *n* (%)	172 (65.9)	92 (80.0)	80 (54.8)	**<0.001**
Brain CT, *n* (%)	94 (36.0)	52 (45.2)	42 (28.8)	**0.009**
Cardiology Consultation, *n* (%)	83 (31.8)	53 (46.1)	30 (20.5)	**0.001**
**Routes of Administration in the ED**				
Oral, *n* (%)	71 (27.2)	4 (3.5)	67 (45.9)	**<0.001**
Intramuscular, *n* (%)	5 (1.9)	0	5 (3.4)	0.121
Intravenous, *n* (%)	129 (49.4)	81 (70.4)	48 (32.9)	**<0.001**
**Pharmacological Treatments in the ED**				
CCB, *n* (%)	24 (9.2)	1 (0.9)	23 (15.8)	**<0.001**
RAS Inhibitors, *n* (%)	58 (22.2)	3 (2.6)	55 (33.7)	**<0.001**
Doxazosin, *n* (%)	3 (1.1)	0	3 (2.1)	0.336
Labetalol, *n* (%)	54 (20.7)	34 (29.6)	20 (13.7)	**0.003**
Diuretics, *n* (%)	41 (15.7)	27 (23.5)	14 (9.6)	**0.004**
β-blockers, *n* (%)	17 (6.5)	13 (11.3)	4 (2.7)	**0.011**
α1-adrenergic receptor antagonists, *n* (%)	3 (1.1)	3 (2.6)	0	0.168
Nitrates, *n* (%)	55 (21.1)	36 (31.3)	19 (13.0)	**0.001**
Anxiolytics, *n* (%)	7 (2.7)	0	7 (4.8)	**0.046**
**Outcomes**				
Inpatient Admission, *n* (%)	131 (50.2)	107 (93.0)	24 (16.4)	**<0.001**
Short Stay Unit, *n* (%)	15 (5.7)	2 (1.7)	13 (8.9)	**0.028**
Discharge, *n* (%)	95 (36.4)	3 (2.6)	92 (63.0)	**<0.001**
Discharge AMA, *n* (%)	20 (7.7.)	3 (2.6)	17 (11.6)	**0.013**
Inhospital Death, *n* (%)	10 (3.8)	10 (8.7)	0	**0.001**

Abbreviations: *n*—number; HE—hypertensive emergency; HU—hypertensive urgency; ED—emergency department; SBP—systolic blood pressure; DBP—diastolic blood pressure; eGFR—estimated glomerular filtration rate; ECG—electrocardiogram; CT—computed tomography; CCB—calcium channel blockers; RAS—renin–angiotensin system; AMA—against medical advice. Continuous data are presented as mean ± standard deviation or median (interquartile range) in case of skewed distribution. Categorical variables are summarized as relative frequencies. A *p*-value < 0.05 was considered statistically significant (bold font).

**Table 2:** Supervised machine learning (ML) analysis which features information gain (IG) and multivariate logistic regressions ascertaining the effects of clinical predictors on the presence of acute hypertension-mediated organ damage (A-HMOD) in patients presenting to the emergency department (ED) with acute hypertensive disorders.

Feature	IG	Adjusted OR (95%CI)	*p*-Value
Troponin I *	10.2%	2.819 (1.649–4.820)	**<0.001**
SBP	8.0%	1.022 (1.003–1.041)	**0.021**
Age	7.8%	1.043 (1.006–1.081)	**0.022**
Creatinine	7.4%	0.805 (0.473–1.369)	0.424
eGFR	6.9%	0.982 (0.953–1.013)	0.251
Herat Rate	4.8%	0.997 (0.978–1.015)	0.724
DBP	4.0%	1.019 (0.990–1.049)	0.193
Male Sex	1.1%	0.548 (0.268–1.122)	0.100
Diabetes Mellitus	0.9%	0.773 (0.349–1.713)	0.526
Coronary Artery Disease	0.8%	0.474 (0.202–1.114)	0.087
Hypertension	0.8%	2.062 (0.873–4.870)	0.100
Chronic Kidney Disease	0.5%	0.941 (0.182–4.876)	0.942

Abbreviations: SBP—systolic blood pressure; eGFR—estimated glomerular filtration rate; DBP—diastolic blood pressure. OR—odds ratio; 95%CI—95% confidence interval. * For binary logistic regressions, troponin I levels were categorized according to the following thresholds: ≤0.1 ng/mL (undetectable or within reference range), >0.1 to <0.3 ng/mL (elevated but below the upper reference limit), and ≥0.3 ng/mL (above the upper reference limit defined by the hospital laboratory). These categories were used to reflect clinically meaningful strata of troponin elevation.

## Data Availability

The data supporting the findings of this study are available from the corresponding authors upon reasonable request due to privacy/ethical restrictions.

## Supplementary Materials

The following supporting information can be downloaded at: https://www.mdpi.com/article/10.3390/diagnostics15162062/s1, Figure S1: Receiver operating characteristic (ROC) curve showing the predictive performance of troponin I for identifying hypertensive emergencies.
