# Supplementary material for: Predictive Modeling of Acute Hypertensive Disorders in a Real-World Cohort: Integrating Clinical Predictors and Data-Driven Methods
Source: Diagnostics (Basel). 2025 Aug 18;15(16):2062. doi: 10.3390/diagnostics15162062 (PMC12386053; doi:10.3390/diagnostics15162062)
Supplement: Supplementary file 1 [file diagnostics-15-02062-s001.zip › diagnostics-3803706-supplementary.pdf]

Predictive Modeling of Acute Hypertensive Disorders:

A Real-World Study.

Ilaria Fucile [1], Filomena Liccardi [2], Maria Virginia Manzi [1], Maria Lembo [1],

Christian Basile [3,4], Orlando Santucci [1], Stefania Auciello [2], Mauro Maniscalco [5,6],

Giorgio Alfredo Spedicato [7], Carmine Morisco [1], Raffaele Izzo [1], Nicola De Luca [1],

Pasquale Ambrosino [8,*], Costantino Mancusi [1,*], Giovanni Esposito [1], Fiorella Paladino [2]

**Corresponding Authors*

[1] Hypertension Research Center & Department of Advanced Biomedical Science, Federico II University, Naples, Italy;
[2] Emergency Department, Antonio Cardarelli Hospital, Naples, Italy;
[3] Department of Clinical Science and Education, Karolinska Institutet, Stockholm, Sweden;
[4] ANMCO Research Center, Heart Care Foundation, Florence, Italy;
[5] Istituti Clinici Scientifici Maugeri IRCCS, Pulmonary Rehabilitation Unit of Telese Terme Institute, Italy;
[6] Department of Clinical Medicine and Surgery, Federico II University, Naples, Italy;
[7] Department of Statistics and Quantitative Methods, Milano-Bicocca University, Milan, Italy;
[8] Istituti Clinici Scientifici Maugeri IRCCS, Scientific Directorate of Telese Terme Institute, Italy.

Table of contents

**Supplemental Figure 1**	**Receiver operating characteristic (ROC) curve showing the predictive performance of troponin I for identifying hypertensive emergencies.**

Supplemental Figure 1. Receiver operating characteristic (ROC) curve showing the predictive performance of troponin I for identifying hypertensive emergencies.

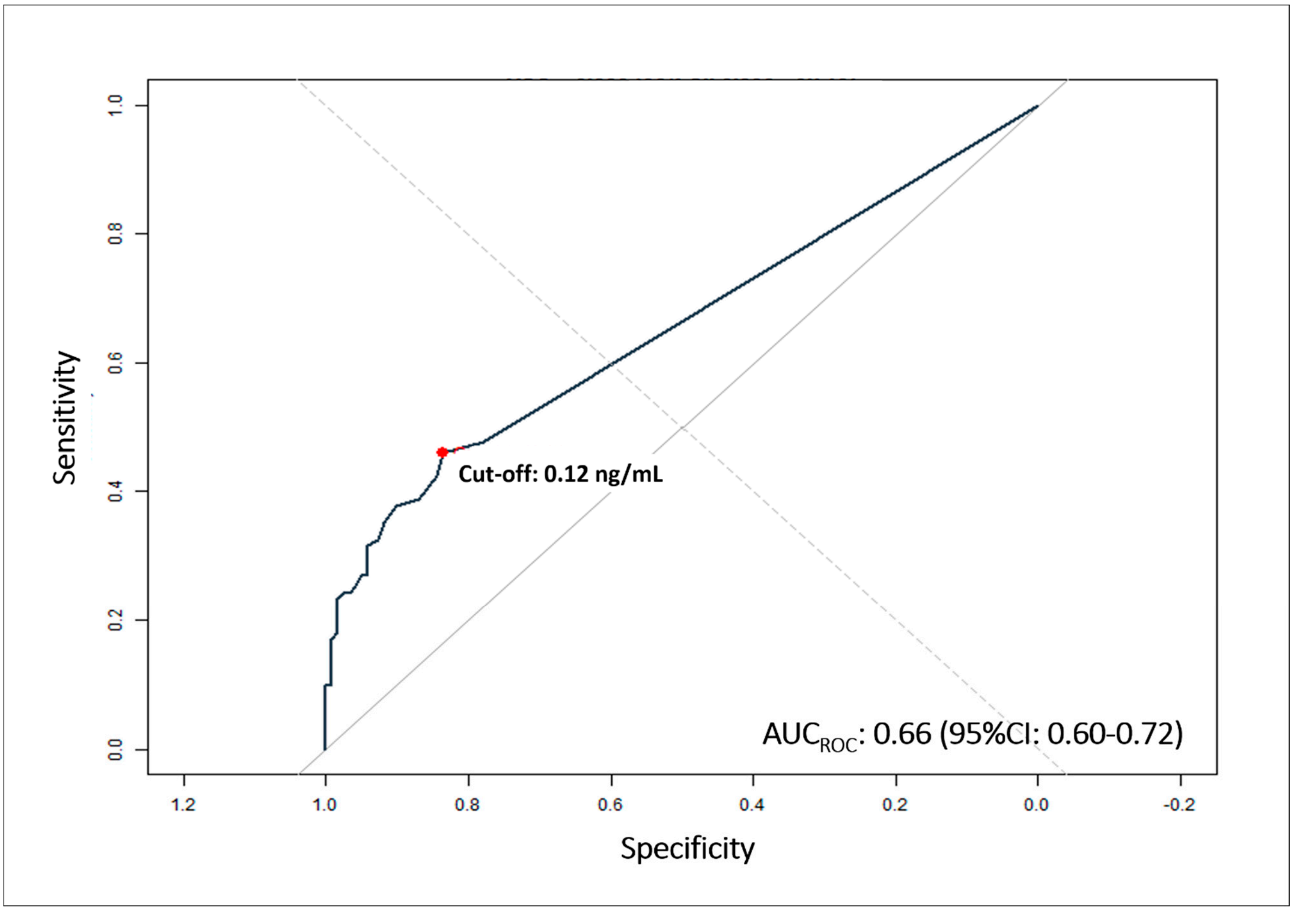

The area under the curve (AUC) is presented with 95% confidence interval (CI). The optimal threshold was determined using Youden's index, which identifies the point maximizing the sum of sensitivity and specificity.
